# Integration of postpartum care into child health and immunization services in Burkina Faso: findings from a cross-sectional study

**DOI:** 10.1186/s12978-018-0602-8

**Published:** 2018-10-11

**Authors:** Danielle Yugbaré Belemsaga, Anne Goujon, Aristide Bado, Seni Kouanda, Els Duysburgh, Marleen Temmerman, Olivier Degomme

**Affiliations:** 1 Département Biomédical et santé publique, Institut de Recherche en Sciences de la Santé (IRSS), 03 B.P 7192, Ouagadougou 03, Burkina Faso; 2 Wittgenstein Centre for Demography and Global Human Capital (IIASA, VID/OAW, WU), Vienna, Austria; 3 International Centre for Reproductive Health, Faculty of Medicine and Health Sciences Department of Uro-Gynaecology, Ghent University, Ghent, Belgium; 4 African Institute of Public Health, Ouagadougou, Burkina Faso; 5 Centre of Excellence in Women and Child Health, Aga Khan University, Nairobi, Kenya

**Keywords:** Postpartum, Maternal and infant health, Integration of services, Burkina Faso

## Abstract

**Background:**

The *Missed Opportunities for Maternal and Infant Health (MOMI)* project, which aimed at upgrading maternal and infant postpartum care (PPC), implemented a package of interventions including the integration of maternal PPC in infant immunization services in 12 health facilities in Kaya Health district in Burkina Faso from 2013 to 2015. This paper assesses the coverage and the quality of combined mother-infant PPC in reproductive, maternal, newborn and child health services (RMNCH).

**Methods:**

We conducted a mixed methods study with cross-sectional surveys before and after the intervention in the Kaya health and demographic surveillance system. On the quantitative side, two household surveys were performed in 2012 (*N* = 757) and in 2014 (*N* = 754) among mothers within one year postpartum. The analysis examines the result of the intervention by the date of delivery at three key time points in the PPC schedule: the first 48 h, days 6–10 and during weeks 6–8 and beyond. On the qualitative side, in depth interviews, focus group discussions and observations were conducted in four health facilities in 2012 and 2015. They involved mothers in the postpartum period, facility and community health workers, and other stakeholders. We performed a descriptive analysis and a two-sample test of proportions of the quantitative data. The qualitative data were recorded, transcribed and analysed along the themes relevant for the intervention.

**Results:**

The findings show that the WHO guidelines, in terms of content and improvement of maternal PPC, were followed for physical examinations and consultations. They also show a significant increase in the coverage of maternal PPC services from 50% (372/752) before the intervention to 81% (544/672) one year after the start of the intervention. However, more women were assessed at days 6–10 than at later visits. Integration of maternal PPC was low, with little improvements in history taking and physical examination of mothers in immunization services. While health workers are polyvalent, difficulties in restructuring and organizing services hindered the integration.

**Conclusion:**

Unless a comprehensive strategy of integration within RMNCH services is implemented to address the primary health care challenges within the health system, integration will not yield the desired results.

## Résumé

**Contexte:**

Le projet de recherche *MOMI* sur les opportunités manquées pour la santé de la mère et de l’enfant, qui avait pour but d’améliorer les soins postpartum (SPP) de la mère et de l’enfant, a mis en œuvre un paquet d’interventions, incluant l’intégration des SPP maternels dans les services de vaccination infantile dans 12 formations sanitaires du district sanitaire de Kaya au Burkina Faso de 2013 à 2015. Cet article évalue l’utilisation et la qualité des SPP du couple mère-enfant dans les services de santé reproductive, maternelle, néonatale et infantile (SRMNI).

**Méthodes:**

Nous avons effectué des enquêtes transversales avec des méthodes mixtes avant et après l’intervention dans le site de surveillance démographique de Kaya. Deux enquêtes ménages ont été réalisées en 2012 (*N* = 757) et en 2014 (*N* = 754) auprès des mères au cours de l’année suivant l’accouchement. L’intervention est évaluée en fonction de la date d’accouchement: premières 48 heures, jours 6–10 et semaines 6–8 et plus. Des entretiens approfondis, des discussions de groupe et des observations ont été menés dans quatre formations sanitaires en 2012 et 2015. Nous avons effectué une analyse descriptive et un test des proportions à deux échantillons des données quantitatives et une analyse du contenu des données qualitatives.

**Résultats:**

Les résultats montrent que les directives de l’OMS, en termes de contenu et d’amélioration des SPP maternels, ont été suivies pour l’administration d’examens physiques et de conseils. Ils montrent également une augmentation significative de la couverture des SPP maternels de 50% (372/752) avant l’intervention à 81% (544/672) un an après le début de l’intervention. Cependant, plus de femmes ont été examinées aux jours 6–10 que lors de visites ultérieures. L’intégration des SPP maternels dans les services de vaccination était faible, avec peu d’amélioration de l’historique médicale et de l’examen physique. Bien que les agents de santé soient polyvalents, des difficultés de restructuration et d’organisation des services ont entravé l’intégration.

**Conclusion:**

L’intégration ne produira les résultats escomptés que si une stratégie globale d’intégration au sein des services de SRMNI est mise en œuvre pour relever les défis des soins de santé primaires dans le système de santé.

## Plain english summary

Maternal mortality and morbidity rates are still substantial in many parts of the world and particularly in sub-Saharan Africa. Several context-specific intervention packages to improve postpartum care (PPC) for both mother and infants have been designed to remedy this problem. In this paper, we study an intervention package that included the integration of maternal PPC in infant immunization services in 12 health facilities in Kaya health district in Burkina Faso from 2013 to 2015. We assess the coverage and the quality of the combined maternal-infant PPC.

We conducted quantitative and qualitative cross-sectional studies in Kaya health district before the start of the intervention in 2012 and 1 year after the implementation in 2014–2015. We analysed the result of the intervention at three key time points in the overall national PPC schedule. Furthermore, we conducted interviews, group discussions and observations within four rural and urban health facilities.

The findings show an improvement of maternal PPC physical examinations and consultations, together with an increase in the coverage of maternal PPC from 50% before the intervention to 81% after the intervention. However, more women were checked up at days 6–10 than at the later stages of the PPC schedule when maternal PPC (history taking and physical examination) was neglected in immunization services. Difficulties in restructuring, in organizing services and collaboration within services hindered the integration. We therefore conclude that integration will not bring the desired results unless a comprehensive strategy of integration within maternal and child health services is implemented.

## Background

Postpartum care (PPC) encompasses the management of mothers, infants and newborns during the period up to 6 weeks after childbirth. Since the World Health Organization (WHO) revised the definition of maternal death to now include late maternal death, late PPC concerns the period from the sixth week to 1 year after delivery [1, 2]. In this study “we define the postpartum period as the period that starts immediately after the birth of the baby and extends up to one year after birth, and PPC as the care provided for mother and infant during this period” [3]. Yet most maternal deaths take place in the immediate postpartum period and most infants deaths occur in the first week [1, 4].

In Burkina Faso, for instance, both maternal and neonatal mortality are high, with 330 maternal deaths per 100,000 live births and 23 neonatal deaths per 1000 live births [5]. Although the postpartum period is a critical stage in the life of mothers and infants, it remains neglected within the reproductive, maternal, newborn and child health (RMNCH) services [4, 6, 7]. The WHO guidelines for maternal and infant PPC include guidance on routine care, such as monitoring the wellbeing of the mother and/or baby, early detection and management of complications, preventive measures and counselling, as well as family planning [4]. The recommended timing, including the number of postpartum contacts for all mothers and newborns, evolved from the renowned formula of “6 hours, 6 days, 6 weeks and 6 months” after birth to four postpartum visits: on the first day, at day 3 (48–72 h), between days 7–14, and 6 weeks after birth. [4, 8]. The timely availability and delivery of PPC for both mothers and newborns is a missed opportunity in RMNCH services in sub-Saharan Africa, as mothers tend to bring their children to the health facility for immunization purposes but do not receive PPC [7]. While research has shown that some background factors hinder the use and usability of PPC, such as low education levels of mothers, beliefs, cultural and religious practices, a lack of resources, as well as the low quality of services, the lack of integration of PPC services for both infants and mothers has also been demonstrated to weaken maternal postpartum (PP) utilization [9, 10]. However, there are several successful cases of integrated intervention with immunization, for instance the distribution of vitamin A and/or of mosquito nets and antenatal care [7, 10–13]. Hence, the coverage, the perceived and actual quality of integrated maternal-infant PPC should get more attention. It is also critical to support the mother–baby relationship and to consider maternal and neonatal health programmes as an integrated whole. Progress could be accelerated by scaling up integrated packages of essential interventions across the continuum of care for RMNCH [14, 15]. In our research, we use the WHO definition of integrated care: “The management and delivery of health services so that clients receive a continuum of preventive and curative services, according to their needs over time and across different levels of the health system” [16]. The premise is that integrating maternal PPC in infant immunization services should increase the use of maternal PPC [17, 18].

The Missed Opportunities for Maternal and Infant Health (MOMI) project, which started in 2011 and ended in January 2016, aimed at improving RMNCH. Facility and community interventions were designed, implemented and assessed in one district per study country (Burkina Faso, Kenya, Malawi, and Mozambique) [19]. The present study focuses on the Kaya health district in Burkina Faso. In this district, a package of interventions was implemented from September 2013 to December 2015, with some pre-intervention activities already having started in July 2013.

This paper aims to assess the outcome of the implemented package of PP interventions by focusing on the integration of PPC in infant immunization services [20]. This integration was designed to be implemented for key interventions at a specific time in the continuum of PPC and was based on the benefits of reorganizing existing structures [17]. The analysis looks at the coverage and the quality of combined mother-infant PPC in RMNCH services by comparing the situation before and after the intervention with a focus on early integration opportunities after delivery in infant immunization services.

## Methods

### Context

The study sites were MOMI project sites in the Kaya health district in the Centre-Nord health region of Burkina Faso. The Kaya district has 564,867 inhabitants and 52 primary health facilities [21]. The MOMI project was implemented in 12 health facilities, including the Kaya Health and demographic surveillance system (Kaya HDSS).

### MOMI baseline assessment and interventions

The MOMI baseline assessment showed that skilled health personnel in the Kaya health district attended 77% of births. Within 7 days after delivery, only 19% of women received maternal PPC, while all newborns received the BCG vaccination. Maternal PPC was poorly integrated within child immunisation and family planning (FP) services [3]. The identifiable factors hindering maternal PPC were the poor organisation of services and quality of care, issues related to understaffing and high staff turnover, a lack of knowledge about maternal health in the community, and traditional beliefs and practices [3].

The MOMI baseline assessment was carried out by local stakeholders and founded on the identification of problems and causes, then using system thinking approaches to specifically review RMNCH policies [3]. Based on this assessment, a tailored package of interventions to strengthen postpartum care delivery was selected and implemented, including: (1) upgrading the quality of immediate PPC provided at the health facilities, with a focus on the detection and management of postpartum haemorrhage and sepsis, (2) supporting mothers and infants during the PP period with the help of female community health workers (CHWs)^1^ and (3) the integration of maternal PPC in infant immunization services [3]. While infant PPC is the overarching anchor for the integration of maternal and child PPC, integration could also happen the other way around, i.e. infant PPC integrated in maternal PP services.

### Intervention design and implementation

Integrated mother-infant PPC in infant immunization services was provided within the health system organization on three occasions [22]. Firstly, at days 6–10, newborns are immunized against the Bacillus Calmette-Guérin virus (BCG) and receive the 0 dose of the poliomyelitis vaccine if they did not receive it before. During this visit, the mother is examined for signs of PP haemorrhage, sepsis, and anaemia. She receives information on the prevention, management and treatment of PP disorders for herself, and on the detection of warning signs in newborns.

Secondly, at weeks 6–8, the infant is immunized against diphtheria, tetanus, pertussis, poliomyelitis, haemophilus influenza type B, rotavirus and pneumococcal using dose 1 conjugate vaccine/ (Penta + Hep 1). At the same time, maternal PPC focuses on the detection, management and treatment of postpartum anaemia, and on advising and providing postpartum family planning. The mother is also informed about the detection of warning signs in infants.

Thirdly, at month 9, the infant is vaccinated against measles and yellow fever. The content of maternal PPC is the same as at the weeks 6–8 visit. We were not able to assess PPC at month 9 due to the lack of information in the registers and *in the household surveys* about this visit [23]. Instead, on top of the intervention at days 6–10, we consider the integrated maternal PPC visit at days 11–41 – between days 6–10 and weeks 6–8 –, and days 42–90, which can be seen as a prolonged weeks 6–8. For the child, we also check for PPC at days 0–5 and days 11–60.

Moreover, as part of the strategy to improve immediate PPC, the newborn should receive the BCG vaccine during the first 48 h after delivery and before leaving the health facility. Therefore, we added the first 48 h to the analysis in order to consider immunization sessions provided during this period and the immediate PPC.

The intervention implementation included several activities. Working sessions were performed in each facility with facility health workers (FHWs) in July 2013 in order to understand the existing level of integration. At the same time, FHWs and managers of primary health facilities, maternity wards and the Expanded Program of Immunization (EPI) were invited to workshops where the MOMI project and interventions were presented. In September 2013, FHWs received training on PPC, including the integration of maternal and infant care. Effective implementation started in October 2013. Staff training (several rounds) continued in December 2013 and in March 2015. Protocols and checklists to support the integration of services were developed, distributed and explained to FHWs from January to February 2014. Quarterly supervision of MOMI activities using a guide was performed from October 2013 to December 2015.

### Study design

As the study is conducted through the MOMI project, which is a case study, the study design follows its global configuration [3]. This present paper was designed around the comparison of the situation prior to and after 1 year of intervention, using a cross-sectional mixed methodology. The survey before the intervention data (including both quantitative and qualitative data) collection took place from December 2012 to May 2013. Another round of quantitative data collection occurred from August to December 2014, within 1 year after the start of the intervention. Qualitative interviews were conducted in 2015 in the framework of MOMI’s final evaluation and were compared with the data collected before the intervention.

### Quantitative survey

The quantitative surveys aimed to investigate the coverage and the content of the pair mother infant PPC and the integration in infant clinics, notably immunization services. The household surveys were carried out before and after 1 year of intervention in the Kaya HDSS, located in the Centre-Nord region of Burkina Faso. The Kaya HDSS contains 7 urban and 18 rural zones within a radius of 20 km, comprising 7 of the 12 primary health facilities included in the MOMI project [24]. The Kaya HDSS 2012–2013 routine household data collection – over a period of 6 months – covered 10,629 households, including 16,801 women of childbearing age, 326 pregnant women and 800 infants. The number of pregnant women is low, probably due to under declaration in the first trimester of pregnancy.

### Sampling technique

We performed a multistage sampling technique, which included data extraction and systematic sampling *with a random start -per health facility and village.*

The data were extracted from Kaya HDSS, pregnancy and delivery –for mothers– and birth records –for infants. The Kaya HDSS data collection and monitoring method is described in detail in Kouanda et al. [24]. We briefly identified all women who had given birth or who were in the last months of pregnancy throughout the Kaya HDSS.

For the survey before the intervention, we selected a systematic sample of 840 mothers in their first-year of PP with an accuracy of 100% from the afore-mentioned database. Eight enumerators performed the data collection using personal digital assistants –small mobile/tablet handheld devices that provide computing and information storage and retrieval capabilities– from December 2012 to January 2013. The survey was specific to the Kaya HDSS.

For the separate survey after 1 year of intervention, we selected a systematic sample of 880 mothers in their first-year of PP out of the 2014–2015 Kaya HDSS household newborn and pregnant women database, following the same procedure as in the survey before the intervention. Ten enumerators carried out the data collection using personal digital assistants and tablets within the Kaya HDSS routine data collection from August 2014 to February 2015.

One sample of mothers in their one-year PP were interviewed before (757 out of 840: 90%) and another one after 1 year of intervention (754 out of 880: 86%) (Table 1). After applying exclusion criteria, there were 732 mothers in the survey before 2012–2013 and 705 in the survey after 2014. Furthermore, thirty three women assessed during the second survey were found to have delivery dates that preceded the intervention and were moved to the sample before the intervention. The survey is not a longitudinal survey, and the mothers are therefore not the same in the two samples. All the surveys were preceded by staff training, pre-testing, and pilot surveys.

**Table 1:** Data included by surveys

Data	Survey before the intervention (2012–2013)	Survey after one year of intervention (2014)	
	in absolute numbers	in absolute numbers	
Sample	840	880	
Data collected	754 (90%)	757 (86%)	
Data excluded by infant year of birth	7 (born in 2010)	2 (born in 2011)	
		16 (born in 2012)	
Missing data on date of birth	15	34	
Data included	732	705	
Data analysed by infant date of birth	Before		After
Infant date of birth	2011–2012	January to June 2013	July to December 2013 and 2014
Data by survey and infant date of birth	732	33	672
Data analysed by period before and after	751		672

We used the same questionnaire on PPC for mother and infant before and after 1 year of intervention, which we complemented with additional information from the Kaya HDSS routine data, such as individual characteristics and household socio-economic characteristics. The latter, including durable assets, housing characteristics and ownership, access to utilities and infrastructure, was used to calculate socio economic quintiles. These allowed us to assess the comparability of the two samples of mothers before and after 1 year of intervention [10].

The questionnaire developed by the researchers included queries about pregnancy outcomes, frequency and content of visits to PP services for maternal and infant immunization. The timing of the interventions that entered the analysis was for maternal PPC: first 48 h, days 6–10, days 11–44 and days 45–90; for infants, it was days 0–5, days 6–10 and days 11–60.

We used cross-tables to study the proportion of mothers who had PP visit(s) during the first 48 h, at days 6–10, at days 11–44 and at days 45–90; and those whose infants had PP visit(s) at days 0–5, days 6–10 and days 11–60. The content of the PPC of the mother-infant pair (history taking, physical exam, consultations) targeting warning signs, detection and management of three main disorders in Burkina Faso (haemorrhage, sepsis, anaemia) and on the integration of services, were used as independent variables.

We analysed the data using the infant’s date of delivery to determine whether the mother and infant were likely to have benefited from the intervention or not. This resulted in two periods (Table 1): 1) before the implementation of the intervention (in 2011–2012 and from January to June 2013), and 2) one-year after the implementation (from July to December 2013 and all of 2014). Frequencies and cross-tabulations were used at the bivariate level to analyse the distribution of the dependent variables by the content of visits. We ran a Chi-Square Test of Pearson as well as a two-sample test of proportions on the before-after linked data in order to determine whether there were differences in maternal and child PPC use before and after the implementation of the intervention.

We performed descriptive and optimal scaling methods for multivariate categorical data analysis using Stata Statistical Software: Release 15 (College Station, TX: StataCorp LLC).

### Qualitative studies

The qualitative studies explored the changes in PPC with the implementation of the interventions from the point of view of the main stakeholders. It aimed to explain the numbers provided by the quantitative surveys and to complement the evaluation. Qualitative study recruitment was based on data saturation in four primary health facilities areas (two rural and two urban) before and during the MOMI project final evaluation study. In addition, the purposive sampling of four contrasting cases by level of implementation (high, low) was done during the evaluation study [25]. The number of CHWs and FHWs before and after did not affect the findings. Before the intervention, data were collected in January 2013 (A). The final evaluation data were collected in July–August 2015 (B).

The data collection involved in depth interviews with health workers in the facilities (A, B) and communities (A, B), key informants (A, B) and women in postpartum period (B) (Table 2). Before the intervention, focus group discussions were conducted with women who had experienced childbirth and with mothers in the PP period. Those were not reproduced in the MOMI final evaluation survey whereby fewer women were included in A than in B. No respondents were interviewed before as well as after the intervention: both panels are composed of different individuals.

**Table 2:** Interview participants in the qualitative studies before and after the intervention

Participants	Women	CHWs	FHWs	Key informants
Before the intervention (B)	42^a^	4	8	8
After the intervention (A)	13	14	16	7

^a^Four focus group discussions were conducted with 42 mothers (in groups of 8–12 women) in PP period (*n* = 22) and women with childbirth experience (*n* = 20)

Furthermore, during the final evaluation phase, we used direct observation by following FHWs while they were delivering services such as routine PPC for mothers and infants, in infant clinics and in family planning services. Sixteen observations reports were written by three MOMI researchers in the four health facilities over a period of 2 weeks.

The data were collected by qualitative research assistants using a guide developed for that purpose. Interviews with women focused on their individual experiences and explored their views on the need for PPC and on the barriers to or facilitators for receiving PPC. With FHWs, CHWs and other stakeholders, the interviews were centred around their views on the importance and provision of PPC, and contextual factors. The data were recorded and transcribed using NVIVO software. The content of the interviews were analysed along both surveys themes. The coding system was developed and implemented by one qualitative researcher for each survey and the consistency was checked a posteriori by the qualitative team members.

The interviews were analysed to determine the opinions of participants on the overall changes in PPC (baseline status, changes in PPC, MOMI project contribution, enablers of PPC uptake) and on the integration of PPC (integrated services and barriers to integrate mother PPC into infant immunization services).

## Results

### Characteristics of participants

#### Quantitative study

The demographic characteristics of the two samples, before and after 1 year of intervention, were assessed as to ensure resemblance and comparability. This is shown in Table 3. The mothers’ characteristics are largely similar, for instance in terms of place of residence, education levels and religion. There is a difference in the age of mothers and the number of children between the two samples. In the survey sample 1 year after the intervention, the women tend to be younger and have fewer children than in the survey before the intervention. This introduces a bias in the results, as a previous study has shown that young mothers with low parity (1–2 children) are more likely to attend PPC [10]. We discuss this in the limitations of the study.

**Table 3:** Comparative table of the characteristics of mothers (before and after)

Characteristics	Before		After		*P*-value
	No.	%	No.	%	
Living areas					
Urban	395	53	392	53	0,993
Rural	351	47	348	47	
Total	746	100	740	100	
Mother’s age					
< =19	41	5	70	9	0.018**
20–29	383	51	372	49	
30–34	191	25	163	22	
> =35	142	19	149	20	
Total	757	100	754	100	
Number of children					
1–2	280	37	325	43	0.001***
3–4	301	40	324	43	
5–6	145	20	86	11	
≥ 7	31	4	19	3	
Total	757	100	754	100	
Mother’s level of education					
None	586	77	554	73	0.13
Primary school	119	16	146	19	
Secondary plus	52	7	59	8	
Total	757	100	754	100	
Religion					
Muslim	633	84	655	87	0.175
Catholic	108	14	89	12	
Protestant	16	2	8	1	
Total	757	100	754	100	
Ethnic					
Moaga	696	92	707	93	0.323
Other	61	8	51	7	
Total	757	100	754	100	

Note: *p-value< 0,05; **p-value< 0,01; ***p-value< 0,001

#### Qualitative study

The interviewed participants in both samples were mostly women; men were more common among HWs and key informants. Most mothers were between 21 and 30 years of age. They had mainly 4 children or more in the final evaluation and fewer than three before the intervention.

### Analysis

#### Quantitative study

We compared the two databases by the infant’s date of birth before the implementation of the intervention and after 1 year of intervention in order to analyse its effects.

Since the *p*-value of Chi-Square Test of Pearson is less than our chosen significance level α = 0.01, we can reject the null hypothesis and conclude that there is an association between the PP visit and intervention implementation (Pearson chi2 (1) = 153). The main results of the quantitative study are presented in Table 4, which shows the share of mothers and infants who benefitted from one PP visit – at any time during the continuum of care before and after 1 year of intervention implementations. The increase is significant: the coverage of maternal PPC increased by 31%, from 50% of women receiving PPC before to 81% afterwards. The uptake is significant in both areas of residence particularly in rural areas from 37 to 76%. The share of infants who had infant PP visits was already high beforehand, at 92% and it increased significantly to 98% afterwards.

**Table 4:** Maternal and infant PP visit by infant year of birth before and after the intervention

	Frequencies n/N (%)		
	Before	After	Pearson chi2 (p)
Mother PP visit	372/751 (50%)	544/672 (81%)	153 (0,000)
Urban	242/404 (60%)	262/303 (86%)	60 (0.000)
Rural	130/347 (37%)	282/369 (76%)	111 (0.000)
Infant PP visit	696/759 (92%)	656/672 (98%)	24 (0,000)
Urban	377/405 (93%)	302/303 (100%)	19 (0.000)
Rural	319/354 (90%)	354/369 (96%)	10 (0,002)

**Fig. 1:**
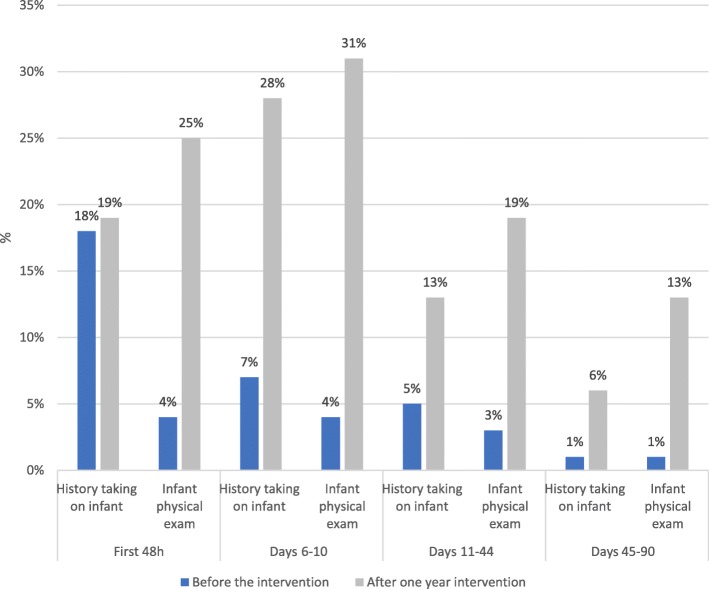
Paediatric history taking and physical examination during maternal PPC before and after one year intervention

We further examined the different episodes of PPC for mothers and infants in order to explore the outcomes of each PP visit. Table 5 describes the mother PP visit before and after 1 year of intervention (firstly at 48 h, at days 6–10 and weeks 6–8, visits at days 11–44 or 45–90), content (history taking, physical examination, consultations), and whether there was an infant examination at the same time, that is of the mother-infant pair. The increase in coverage is visible across the PPC continuum (*P*-value< 0,001). It is particularly important at days 6–10 for maternal PP visits that increased from 21% (154/751) beforehand to 49% (330/672) after 1 year of interventions, although 50% of the latter group did not systematically receive a physical examination during the visit. While the share of women receiving PPC in days 42–90 is lower than in the earlier days after the delivery, the increase is substantial, as the share of women benefitting from PPC at this stage was extremely low before the intervention: 3% (20/751) of women visited PPC services at days 45–90 before the interventions, and 17% (117/672) after the intervention. Although the share of women getting advice on their health and on that of their infant increased significantly, it still lags behind other PPC components, such as physical examination. The combined mother-infant PPC examination seem to be increasingly happening within the visit at days 6–10 –from 4% (28/751) beforehand to 31% (211/672) after 1 year of interventions (Fig. 1).

**Table 5:** Mother PP visits by infant year of birth before and after one year of intervention

Variables	First 48 h % (n)			Days 6–10% (n)			Days 11–44% (n)			Days 45–90% (n)		
	Before (*N* = 751)	After (*N* = 672)	Pearson chi2 (p)	Before (*N* = 751)	After (*N* = 672)	Pearson chi2 (p)	Before (*N* = 751)	After (*N* = 672)	Pearson chi2 (p)	Before (*N* = 751)	After (*N* = 672)	Pearson chi2 (p)
Mother PP visit	28% (213)	40% (268)	157 (0.000)	21% (154)	49% (330)	184 (0.000)	8% (62)	35% (233)	220 (0.000)	3% (20)	17% (117)	196 (0.000)
History taking^a^	27% (200)	39% (259)	159 (0.000)	18% (132)	47% (316)	184 (0.000)	7% (52)	31% (211)	221 (0.000)	2% (16)	16% (105)	197 (0.000)
Physical exam^a^	19% (144)	30% (202)	160 (0.000)	17% (131)	40% (269)	185 (0.000)	7% (54)	30% (204)	214 (0.000)	2% (17)	15% (104)	197 (0.000)
Consultations^a^	5% (38)	20% (134)	184 (0.000)	8% (58)	26% (176)	192 (0.000)	4% (29)	15% (99)	181 (0.000)	1% (8)	7% (49)	196 (0.000)
History taking on infant^a^	18% (132)	19% (128)	166 (0.000)	7% (56)	28% (191)	200 (0.000)	5% (37)	13% (87)	179 (0.000)	1% (8)	6% (38)	197 (0.000)
Infant visit^a^	5% (35)	25% (169)	242 (0.000)	4% (28)	31% (211)	261 (0.000)	4% (22)	19% (129)	181 (0.000)	1% (5)	13% (85)	205 (0.000)

^a^at mother PP visit

We investigate the content of mother PPC to determine the quality of the visit. Table 6 presents the content of mother PP before and after 1 year of intervention for those who had the PP check-up. Before the intervention, history taking mainly focused on general health. After the intervention, women were more likely to be asked about warning signs i.e. general pain (25% before versus 43% after), bleeding (18% before versus 39% after) during the first 48 h and fever (16% before versus 45% after) at days 6–10. At weeks 6–8 PP visit, history taking focused on family planning (26% before versus 35% after at days 11–44). There was an increase in the number of women who had had their temperature (i.e. 52% before and 71% after the first 48 h) or blood pressure checked (58% before and 71% after) the first 48 h, and were assessed for anaemia (26% before and 55% after) the first 48 h. Except in the first 48 h, during which the frequency of vaginal examinations increased (from 47% before to 57% after), the trend in other visits declined. Although the data do not allow for the strict attribution of these results to the intervention, there is still possibility of a linkage, as the protocol did not longer require a vaginal exam during PP visit except for women who were bleeding. The share of PP mothers who are receiving counselling on exclusive breastfeeding and the use of impregnated mosquito nets increased in the first 48 h from 4 to 31% in 2014 (*p* value < 0.001) and from 8 to 31% (*p* value < 0.001). Infant examinations during maternal PPC increased from 16 to 63% in the first 48 h, from 13 to 55% in days 6–10, from 35 to 55%, in days 11–41 and from 25 to 75% in days 42–90.

**Table 6:** Content of mother PP visit before and after one year of intervention

Variables	First 48h			Days 6–10			Days 11–44			Days 45–90		
	Before	After	p	Before	After	p	Before	After	p	Before	After	p
	%, *N* = 213	%, *N* = 268		%, *N* = 154	%, *N* = 330		%, *N* = 62	%, *N* = 233		%, *N* = 20	%, *N* = 117	
History taking^a^	94%	97%	0.000	86%	96%	0.000	84%	91%	0.000	80%	90%	0.000
General health	92%	95%	0.000	83%	91%	0.000	3%	32%	0.000	80%	86%	0.000
Fever	31%	43%	0.000	16%	45%	0.000	15%	27%	0.000	20%	22%	0.000
General pain, headache	25%	43%	0.000	25%	43%	0.000	21%	25%	0.000	10%	17%	0.000
Bleeding	18%	39%	0.000	32%	41%	0.000	21%	25%	0.000	5%	12%	0.000
Family planning	8%	36%	0.000	25%	30%	0.000	26%	35%	0.000	35%	32%	0.000
Physical exam^a^	68%	75%	0.000	85%	82%	0.000	87%	88%	0.000	85%	89%	0.000
Temperature check	52%	71%	0.000	60%	72%	0.000	58%	75%	0.000	65%	82%	0.000
Blood pressure check	58%	71%	0.000	72%	74%	0.000	71%	78%	0.000	75%	85%	0.000
Examination of conjunctiva, tongue, palms	26%	55%	0.000	28%	40%	0.000	15%	28%	0.000	20%	18%	0.000
Vaginal exam	47%	57%	0.000	68%	54%	0.000	65%	43%	0.000	45%	25%	0.000
Consultations^a^	18%	50%	0.000	38%	53%	0.000	47%	42%	0.000	40%	42%	0.000
Bleeding	12%	29%	0.000	25%	33%	0.000	16%	15%	0.000	30%	21%	0.000
Fever	11%	26%	0.000	10%	32%	0.000	15%	15%	0.000	10%	8%	0.000
Sexual intercourse	4%	25%	0.000	3%	15%	0.000	16%	10%	0.000	5%	17%	0.000
Family planning	10%	27%	0.000	25%	30%	0.000	35%	35%	0.000	40%	32%	0.000
Benefits of exclusive breastfeeding	4%	31%	0.000	6%	32%	0.000	3%	26%	0.000	10%	26%	0.000
Keeping baby warm	2%	22%	0.000	1%	19%	0.000	0%	10%	0.000	5%	7%	0.000
Bed net use	8%	31%	0.000	18%	35%	0.000	27%	25%	0.000	5%	12%	0.000
HIV AIDS prevention	8%	16%	0.000	21%	16%	0.000	16%	7%	0.000	5%	12%	0.000
HIV AIDS diagnosis and treatment	3%	17%	0.000	4%	13%	0.000	2%	5%	0.000	0%	10%	0.000
Question on consultation understanding	3%	26%	0.000	5%	33%	0.000	8%	23%	0.000	0%	21%	0.000
Integration												
History taking on infant	62%	48%	0.000	36%	58%	0.000	60%	37%	0.000	40%	32%	0.000
Infant visit	16%	63%	0.000	18%	64%	0.000	35%	55%	0.000	25%	73%	0.000

^a^at mother PP visit on (Total); the frequencies and Chi-Square Test of Pearson results are available on request

We performed a similar analysis to see the effects of the intervention on infant PP visits in terms of coverage and content.

**Fig. 2:**
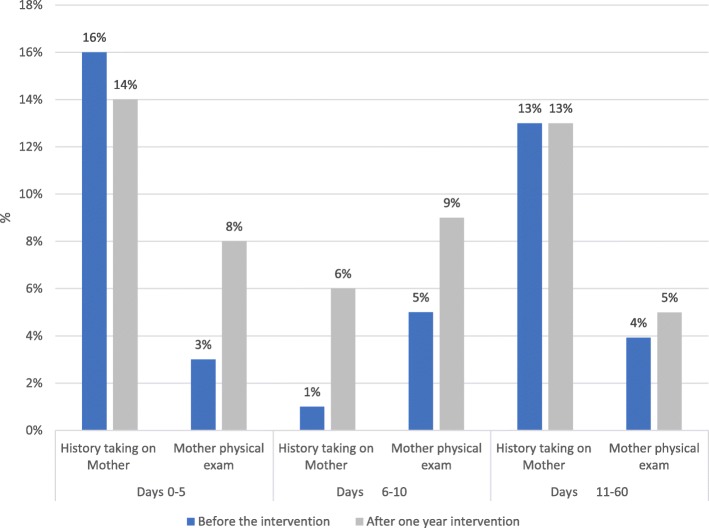
Maternal history taking and physical examination at infant clinics before and after one year intervention

Table 7 shows that infant PP visits service coverage at days 0–5, days 6–10 and days 11–60 increased with the intervention. The results significant *P*-value< 0,001- show little improvements in the physical examination of mothers, thus pointing at a very low integration of maternal PPC within infant clinics. However, more women were questioned for history taking before the intervention than after it (Fig. 2).

**Table 7:** Infant PP visits before and after one year of intervention

Variable	Days 0–5% (n)			Days 6–10% (n)			Days 11–60% (n)		
	Before (*N* = 759)	After (*N* = 672)	Pearson chi2 (p)	Before (*N* = 759)	After (*N* = 672)	Pearson chi2 (p)	Before (*N* = 759)	After (*N* = 672)	Pearson chi2 (p)
Infant clinics	45% (339)	52% (347)	263 (0.000)	16% (122)	33% (225)	74 (0.000)	70% (535)	86% (581)	56 (0.000)
History taking^a^	29% (223)	39% (263)	35 (0.000)	10% (77)	27% (181)	85 (0.000)	28% (216)	42% (285)	65 (0.000)
Physical exam^a^	42% (319)	50% (333)	28 (0.000)	16% (118)	32% (218)	74 (0.000)	69% (525)	84% (566)	57 (0.000)
Consultations^a^	7% (52)	18% (123)	63 (0.000)	1% (7)	11% (71)	102 (0.000)	10% (79)	20% (136)	70 (0.000)
Integration history taking on mother^a^	16% (120)	14% (91)	623 (0.000)	5% (39)	9% (62)	986 (0.000)	13% (102)	13% (88)	107 (0.000)
Integration mother physical exam^a^	3% (25)	8% (52)	36 (0.000)	1% (8)	5% (31)	78 (0.000)	4% (21)	3% (29)	57 (0.000)

^a^at infant PP visit

Table 8 lists the practice in the detailed content of infant physical examination before and after intervention. The analysis shows significant *P*-value< 0,001 – that before the project intervention, women were mostly asked about the general health of their infant, while after the intervention e.g. at days 6–10, they were also asked about the infant’s fever (51%), and their experiences with breastfeeding (45%).

**Table 8:** Content of Infant clinics before and after one year of intervention

Variable	Days 0-5			Days 6-10			Days 11-60		
	Before	After	p	Before	After	p	Before	After	p
	%, *N* = 339	%, *N* = 347		%, *N* = 122	%, *N* = 225		%, *N* = 535	%, *N* = 581	
History taking^a^	66%	76%	0.000	63%	80%	0.000	40%	49%	0.000
General health	63%	76%	0.000	61%	80%	0.000	38%	48%	0.000
Fever	25%	41%	0.000	20%	51%	0.000	15%	31%	0.000
Breastfeeding	23%	40%	0.000	17%	45%	0.000	12%	27%	0.000
Conditions of eyes	9%	29%	0.000	12%	35%	0.000	4%	15%	0.000
Conditions of cord	14%	28%	0.000	14%	31%	0.000	3%	12%	0.000
Physical exam^a^	94%	96%	0.000	97%	97%	0.000	98%	97%	0.000
Temperature check	36%	63%	0.000	44%	71%	0.000	54%	54%	0.000
Weight	34%	50%	0.000	49%	58%	0.000	93%	89%	0.000
Examination of cord conjunctiva, tongue	20%	33%	0.000	28%	36%	0.000	8%	13%	0.000
BCG-polio 0 vaccine	91%	85%	0.000	85%	84%	0.000			
Pentavalent- Hep 1 vaccine							84%	85%	0.000
Consultations^a^	15%	35%	0.000	6%	32%	0.000	15%	23%	0.000
Wet cord with blood/ pus	3%	9%	0.000	3%	12%	0.000	1%	5%	0.000
Feeding difficulty	4%	15%	0.000	2%	17%	0.000	1%	9%	0.000
Swollen tights eyes	2%	9%	0.000	0%	12%	0.000	1%	4%	0.000
Baby feels very cold while hot	2%	16%	0.000	1%	16%	0.000	2%	9%	0.000
Baby feels very hot while undressed	2%	14%	0.000	1%	16%	0.000	2%	9%	0.000
Difficulty breathing	1%	7%	0.000	1%	10%	0.000	0%	3%	0.000
Lethargy	0%	4%	0.000	0%	8%	0.000	0%	2%	0.000
Convulsions	0%	4%	0.000	2%	6%	0.000	0%	2%	0.000
Exclusive breastfeeding	12%	29%	0.000	11%	28%	0.000	13%	19%	0.000
Immunization calendar	10%	30%	0.000	8%	25%	0.000	11%	19%	0.000
Keeping baby warm	1%	11%	0.000	1%	12%	0.000	1%	7%	0.000
Bed net use	9%	22%	0.000	5%	22%	0.000	7%	15%	0.000
Consultation understanding	8%	21%	0.000	7%	17%	0.000	1%	13%	0.000
Integration									
History taking on mother	35%	26%	0.000	32%	28%	0.000	19%	15%	0.000
Mother physical exam	7%	15%	0.000	7%	14%	0.000	4%	5%	0.000

^a^at infant PP visit on (Total); the frequencies and Chi-Square Test of Pearson results are available on request

The same proportion (about 84%) of infants received Penta + Hep1 vaccines at month two (days 11–60) before and after the intervention. Counselling on exclusive breastfeeding and immunization schedules were 28% and 25% respectively, with *P*- value < 0,001 during the neonatal period after the intervention compared to 11% and 8% before it. The proportion of women who were counselled about most warning signs of fever in infants increased by about 10%, at days 6–10 for instance, from 1% before to 16% after the intervention. The share of women who received recommendations in case of difficulties in their infant’s breathing rose from 1 to 10% at days 6–10 PPC. The mother’s understanding of the consultation increased at days 0–5 from 8% before to 21% after the intervention and at days 6–10 from 7 to 17%.

#### Qualitative study

After describing the situation before the intervention, we further investigated the changes that took place, as well as the specific contribution of MOMI’s interventions, before concluding with the analysis of the integration of maternal PPC into infant immunization services.

### Women’s PPC experiences before the intervention

Before the intervention, PPC was neglected by all health providers and all women. Deficiencies were found in the organization, quality, demand and the cost of PPC [10].

*“I think that information are insufficient: a woman does not know she has to come back”* (FGD woman with experience in childbirth, before the intervention).

*“They do not give much importance to PP. Those returning do so if they have concerns. For some, if they have no problems, they do not see the importance of returning for the examination by the HW. They say I’m healthy, my child is fine too, I do not see the importance of returning at day 6 or 42”* (FHW, auxiliary midwife, before the intervention).

Day 42 PPC visit was considered to be exclusively for family planning, and was hence neglected when the woman did not take family planning into consideration.

*“The postpartum appointment for family planning.”* (FGD, woman with experience in childbearing, before the intervention).

### Change in PPC with implementation of the interventions

MOMI project activities, especially training, supervision and sensitization, contributed to improvements in the perception of mothers’ and health workers’ knowledge and motivation [25]. Observations in all settings point to an improvement in the quality of PPC.

*“They say the work of the MOMI project bears fruit, because before it women were not going to the maternity and health centres; but with the MOMI project, we see that women have understood the importance of health centres…with the multiplication of sensitization, they accept and respect the appointment.”* (CHW, final evaluation).

The multiparous women in the PP period provided a comparison of changes in PPC before and afterwards. They corroborate that the improvement in the quality of care, with the early detection and management of warning signs motivates mothers to use PPC services.

*“Before, at the delivery, health workers didn’t tell us to come back at the (6th) sixth day, nor for the 42th day They were only telling us to come for the weighing, but since last year, if you give birth, you are told to return for the 6th and 42th day visits before they start weighing.”* (Woman, final evaluation).

Women are more confident and satisfied with PPC.

*“There is basically a difference between before and now. Now, with PP visits, if you or your baby are not fine, they tell you. .. I am very satisfied with PPC. In the past, maybe you give birth, and after childbirth, you or your child is sick and you don’t realize. But now, after childbirth, you are relieved.”* (Woman, final evaluation).

Women have become more motivated to seek help within PPC facilities.

*“What motivates me is the fact that if I go to the hospital for any pain, I find relief. This is what motivates me to return, because if you go to the hospital, health workers can find a disease and heal you. Or, you may think that you are in good health, but if you go there, they will confirm either way”.* (Woman, final evaluation).

However, PPC at days 42–90 did not improve and was still neglected by women after the intervention.

*“On the 42nd day, it’s carelessness of women themselves, as they believe they have nothing. They have no problem (speaking in local language): why should I return”* (FHW, auxiliary midwife, final evaluation).

### Integration of mother PPC in infant’s immunization services

Primary health services are organized within dispensary and maternity, located mostly in separate buildings. Since FHWs in primary care are supposed to be polyvalent, all the staff, including those from dispensaries, were trained by MOMI to provide PPC.

“*When talking about MOMI we must talk about training. Those are staff capacities strengthened on postpartum care. People really were astonished; they are interested in the problem; we were not able to integrate it at the vaccination. This way, people know the advantages and the danger when women don’t come”.* (FHW, Nurse, final evaluation).

Explanations and clarifications from the project implementers during monitoring and supervision allowed for a better understanding of the intervention and its implementation in health facilities.

*“This varies from one health facility to another; there are health facilities which immediately took it on, but there are others where we had to walk, walk and explain all day long for this to proceed. Until today, there is no health facility clinic into which we have not succeeded in integrating it”.* (Key informant, final evaluation).

The collaboration between primary health facility units, for instance dispensary and maternity, improved with the interventions.

*“« What has changed is the involvement of dispensary and maternity staff. Before, everyone was in his corner, while now everyone is integrated here. …Dispensary staff refer women to maternity and* vice versa*. Before, everyone was on his side. For example, women coming to vaccination were vaccinated and then they went away. We did not check her date of delivery or the postpartum visit. Now, we ask if they have received the postpartum care; if they didn’t receive PPC we accompany them.”* (FHW, Auxiliary Nurse, final evaluation).

PPC level of integration depends on the timing of the visit. At days 6–10, the dyad mother newborn PPC is integrated to BCG immunization services.

*“It is integrated because if the woman comes with her baby to the 6*^*th*^ *or 7*^*th*^ *day visit … we check the immunization schedule and ask her to go to the dispensary for immunization.”* (FHW, nurse, final evaluation).

Integration becomes less efficient as one moves along the continuum of PPC. As a result, maternal services are less well integrated to immunization services particularly at weeks 6–8 of PPC. Firstly, reporting seems to be very time-consuming. Most observers noted that the tools and registers used to fill in took on average ¾ of the visit time.

*“No, we have not integrated it into the vaccination. That takes time. For vaccinations and weight, we check if the infant is up-to-date with his vaccinations and with weighing*. *Otherwise, postpartum most of the time … honestly we don’t check”* (FHW, nurse, final evaluation).

Moreover, observations for the final evaluation report mentioned long waiting times for PPC, although not necessarily longer than before the implementation of the intervention.

*“The waiting is due to affluence, because we have to receive women for infant clinics, family planning, postpartum; it is usually difficult to go fast. The women who come for PP have to do the queuing.”* (FHW, midwife, before the intervention).

This is linked to the lack of staff, which is particularly acute in rural settings, rendering integration difficult.

*“… if there is a mother and child intervention, it means that all women will come, and if staff is down, the number of staff must increase”.* (FHW, auxiliary nurse, before the intervention).

Those interviewed also cited other reasons that limit the success of the integration and that are not directly linked to integration, such as the cost of drugs and supplies. Some women do not pay anything, since FHW use surplus equipment from free services e.g. gloves for deliveries. Others have to pay for supplies, such as speculum and gloves.

*“They have to pay CFA 750 because they have a device to look into the body for things they can’t see with their naked eyes. But if you don’t have CFA 750, there is no trouble, they will examine you but not in-depth. If you have CFA 750, they will check with their tool.”* (CHW, final evaluation).

## Discussion

Our paper studies the effects of the effective PPC uptake for the mother-infant pair in RMNCH services and the quality of care (technically, both in terms of effective delivery of evidence-based practice, and also as perceived by patients). Before the intervention’s implementation, maternal PPC was largely neglected by users who did not see the importance of returning for an examination, as mentioned by an FHW [25].

We found that the coverage of maternal PPC has improved in the Kaya health sub district, possibly due to the synergy of several programme efforts in the area, including the implementation of the MOMI project package of interventions that increased the demand for PPC by sensitizing primary users and community leaders, and developed health workers’ capacity to respond to this demand, thus creating positive synergy [25]. *The combined efforts contributed to generate more important improvements in rural than in urban settings due to the presence of stronger social networks* [26, 27]. All observations point to an improvement in the quality of PPC.

While the integration of maternal care with the delivery of BCG vaccine in days 6–10 was greatly improved, maternal PPC and integrated care were still lagging behind in weeks 6–8. The possible explanations are the major difficulties in the structuring and organization of health facilities, leading to long waiting times for mothers in the context of staff shortage. The paper shows that anchoring maternal PPC to infant immunization services was quite difficult.

This issue can be linked to many factors. Mothers in the PP period still perceive the weeks 6–8 PP visit as exclusively related to postpartum family planning (PPFP) and do not see the need to attend if they do not have FP needs, as also mentioned during focus group discussions. Moreover, previous studies identified that social pressures influence FP utilization rate as common biases in decision-making [25, 28, 29].Sensitization messages need to be refined and should target the dilemma of tracking women for PPFP strategies in PPC at weeks 6–8 in order to improve women and communities’ perceptions about the visit [29].

Some structural issues i.e. staff, organization of PHC units, collaboration within units; prevent the reorganization of health services around integrated PPC. Earlier papers have found that the integration of services is affected by structural barriers or constraints contingent to the health system, in which maternal death are rooted, explaining largely high maternal mortality in the low-middle income countries in sub-Saharan Africa and South Asia [17, 30, 31].

Some constituents of PHC involving the first contact, continuous, comprehensive, and coordinated care contain some dimensions of integrated care [32–34]. The first level of health care organization in Burkina Faso follows the eight primary health care (PHC) components among which maternal and child health, including family planning and immunization against major diseases, are distinct. Immunization is part of the services provided by dispensaries. Primary health care activities occur within both dispensaries and maternities for maternal and infant care, which may not be optimal for integrated RMNCH services, although the staff is polyvalent.

The causal pathway of the intervention was designed through the document review, baseline studies and the involvement of some chosen stakeholders through the policy advisory board [3]. The integration implementation strictly followed the design and the abovementioned guidelines, yet, at the same time, the integration process was not fully detailed [25, 35, 36]. More research is needed to derive programmatic and policy improvements.

Since the health system must be improved and must get political attention, constraints and barriers to integration must be analysed in order to find ways to overcome them [13, 34, 37, 38]. We found some deficiencies in the collaboration within PHC units, whereas it would be essential for the integrated provision of PPC [39, 40]. However, the final evaluation showed that collaboration between dispensaries and maternities had improved. Strengthening collaboration among health facility management units is key to restructuring service flows.

With the development of RMNCH policies, it is important to review the organization of activities in primary health care facilities in order to develop solutions for linking health care for the mother-baby dyad [17, 34, 41]. The organization of primary health facilities in Burkina Faso would need to be enhanced in order to improve RMNCH. Further research on human resources for health should assess the leadership and collaboration mechanisms in the health sector, especially in PHC.

We found out that some women do not pay any fees for PPC, since FHW are using surplus equipment from free services e.g. gloves for deliveries. Others have to pay for supplies, such as speculum and gloves. At the time of the study, there was no clear financing policy for maternal PPC. Yet, subsidies for delivery and obstetric care were in place, as well as free immunization services. Since March 2016, free care for mothers and children under 5 years of age, including the provision of maternal PPC until week 6, was decreed by the president of Burkina Faso [47]. Further research assessing the cost of PPC could provide evidence on the cost at demand and supply side.

We found that the providers training on PPC contributed to improving PPC services. While MOMI tried to tackle process mapping within the interventions, long waiting times for PPC have persisted, mostly due to lengthy reporting procedures and a lack of staff in most health centres, particularly in rural areas. The large implementation of integrated services delivery within flow procedures should induce less (perceived and actual) waiting times for users [40]. This opportunity should be improved by including maternal PPC in infants’ clinics and immunization services [42]. Integration in the context of limited staff and heavy workload would require process mapping to eliminate unnecessary steps and to reduce client waiting time [43, 44]. However the health staff unequal distribution between rural and urban settings and insufficiency in quantity need to be addressed by multi-sectoral actors –ministry of health as well as ministry of education and public administration- [45].

The study suffers from several limitations that mostly emerge from the study’s design, separating the period before the intervention and after the start of the intervention (after one year and at the time of the final evaluation). This design has several flaws that suggest the need for caution regarding the outcome of the evaluation results. The after survey should have been conducted closer to the end of the project after all implementation activities had been routinely in place. Also, we noticed that some women’s background features were not the same in the two samples before and after one year of intervention. However, we felt that the mixed method contributed to increasing the validity of the study [46]. Firstly, in a systematic review that discussed the non-consensus on the randomized controlled studies criteria needed to access the quality of programme evaluations, Atun (2010) asserted the need for robust design in order to evaluate integrated programmes; longitudinal studies, for instance [39]. A time trend analysis of monitored indicators from one year prior to the intervention’s implementation to December 2015 found similar results [23]. The comparability of the findings prevents the recall bias issues in the reporting of the visits, since the interviews were performed many months after the visits took place. Secondly, the before and after survey samples differ somewhat due to the background variables (age of the mother and number of children). This difference could affect the effects of the intervention because differences in results between the period before and after intervention could also be affected by the effect of the structure/composition of the two samples. This bias in the quantitative results was partly dealt with via the mixed approach (quantitative vs. qualitative) of the study. Without overcoming the limitations of the quantitative study, the qualitative analysis somehow confirms its results [46]. However, we will explore and perform multivariable logistic regression for comparative issue in further papers.

Our study’s results cannot be solely attributable to the MOMI project. Indeed, several programmes and projects on the RMNCH coexisted on the study site. Among these, the ViM (Victory against Malnutrition) Project from Save the Children requires women to present their infant health book with proof of all visits, for instance at day 42 PP, in order to benefit from food distribution. A recent study conducted in 10 health districts of the Centre Nord region and the Sahel region, including the Kaya Health district, shows that the Save the Children programme had little impact on the utilization of postpartum services [40]. Performance based financing (PBF)^2^ [41] piloted in Kaya district since March 2014 has motivated FHWs to engage in PPC activities [25]. Indeed, literature shows that PBF contributed to improving the financial and intrinsic motivation of health workers in Sierra Leone, Burundi, Nigeria, and Malawi, for instance [42, 43].

There are some other limitations that have to be kept in mind. Firstly, the period delimitation for mother and infant PPC does not strictly refer to weeks 6–8 PPC but to days 11–41 and 42–90. The timing was developed for the survey prior to the intervention implementation. Secondly, data collected on consultations were limited to individual counselling during PPC. Sensitization within group talks with mothers prior to the visit that took place in the primary health facilities were not measured in our paper. Thirdly, the study, which was based on national protocols, did not collect data on FP utilization, but rather on sensitization to FP [44].

Beyond all of these limitations, we feel that the analysis still makes a valuable contribution to the issue of integrated maternal and infant PPC in the African context.

## Conclusion

This paper highlighted the improvement of PPC coverage and quality within RMNCH services. The delivery of PPC to the mother-infant pair proved successful for visits at days 6–10, but less so in weeks 6–8, and in subsequent visits. The integration of maternal PPC within infant immunization services faced severe challenges. The paper point at the need to research the structural barriers present in the health system, which hindered the implementation of such an intervention e.g. human resources, financing. The reorganization of the PHC into a health system capable of delivering comprehensive and resilient, tailored integrated RMNCH services could be a step forward.

## Abbreviations

BCG: Bacillus Calmette-Guérin
CHW: Community Health Workers
DTPP: Diphtheria, Tetanus, Pertussis and Poliomyelitis
FHW: Facility Health Workers
FP: Family Planning
Kaya HDSS: Kaya Health and Demographic Surveillance System
MOMI: Missed Opportunities for Maternal and Infant Health
PBF: Performance Based Financing
Penta + Hep1: DTP vaccine, haemophilus influenza type B, rotavirus and pneumococcal conjugate vaccine/dose 1 (Penta + Hep 1)
PHC: Primary Health Care
PP: Postpartum
PPC: Postpartum Care
PPFP: Postpartum family planning
RMNCH: Reproductive Maternal Newborn and Child Health

## Footnotes

1 Female CHW were originally village midwives, who now accompany women in child birth to the health facility.

2 Performance based financing is a “strategy for using explicit performance-based subsidies to support the delivery of basic services where policy concerns would justify public funding to complement or replace user-fees. The core of the approach is the contracting out of service delivery to a third party, where payment of public funds is tied to the actual delivery of these services” (p. 1).
